# PACK-CXL: Corneal cross-linking in infectious keratitis

**DOI:** 10.1186/s40662-016-0042-x

**Published:** 2016-04-19

**Authors:** David Tabibian, Cosimo Mazzotta, Farhad Hafezi

**Affiliations:** Laboratory for Ocular Cell Biology, University of Geneva, Geneva, Switzerland; Siena Crosslinking Center, Siena University Hospital, Siena, Italy; Department of Ophthalmology, University of Southern California, Los Angeles, CA USA; EyeCare Laboratory Research Zurich Associates (ELZA) Institute, Webereistrasse 2, 8953 Dietikon, Switzerland; Department of Ophthalmology, Northampton General Hospital, Northampton, United Kingdom

**Keywords:** Infectious Keratitis, Corneal cross-linking, Keratoconus, CXL, PACK-CXL, Corneal ulcer, Ultraviolet light A, Riboflavin

## Abstract

**Background:**

Corneal cross-linking (CXL) using ultraviolet light-A (UV-A) and riboflavin is a technique developed in the 1990’s to treat corneal ectatic disorders such as keratoconus. It soon became the new gold standard in multiple countries around the world to halt the progression of this disorder, with good long-term outcomes in keratometry reading and visual acuity. The original Dresden treatment protocol was also later on used to stabilize iatrogenic corneal ectasia appearing after laser-assisted in situ keratomileusis (LASIK) and photorefractive keratectomy (PRK). CXL efficiently strengthened the cornea but was also shown to kill most of the keratocytes within the corneal stroma, later on repopulated by those cells.

**Review:**

Ultraviolet-light has long been known for its microbicidal effect, and thus CXL postulated to be able to sterilize the cornea from infectious pathogens. This cytotoxic effect led to the first clinical trials using CXL to treat advanced infectious melting corneal keratitis. Patients treated with this technique showed, in the majority of cases, a stabilization of the melting process and were able to avoid emergent à chaud keratoplasty. Following those primary favorable results, CXL was used to treat beginning bacterial keratitis as a first-line treatment without any adjunctive antibiotics with positive results for most patients. In order to distinguish the use of CXL for infectious keratitis treatment from its use for corneal ectatic disorders, a new term was proposed at the 9th CXL congress in Dublin to rename its use in infections as photoactivated chromophore for infectious keratitis -corneal collagen cross-linking (PACK-CXL).

**Conclusion:**

PACK-CXL is now more frequently used to treat infections from various infectious origins. The original Dresden protocol is still used for this purpose. Careful modifications of this protocol could improve the efficiency of this technique in specific clinical situations regarding certain types of pathogens.

## Background

**Fig. 1:**
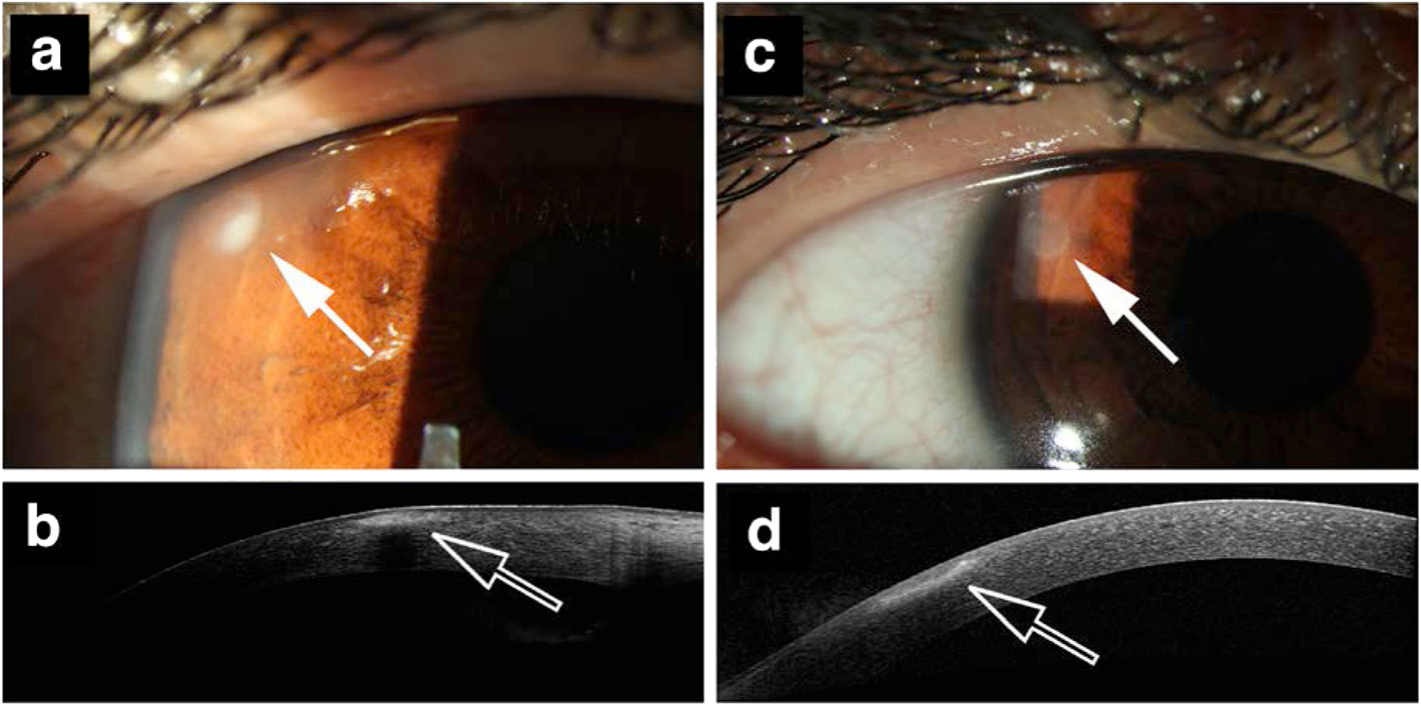
Pre- and post-treatment peripheral infectious keratitis. **a** Anterior segment photography of a patient with early peripheral infectious keratitis (*arrow*). **b** Anterior segment OCT of the lesion. **c** Anterior segment photography of the same patient 7 days after PACK-CXL (9 mW/cm^2^ irradiance for 10 min) with resolution of the peripheral infectious keratitis (*arrow*). **d** Anterior segment OCT of the lesion at day 7 after PACK-CXL

The management of corneal ectatic disorder was completely transformed this past decade through the development and rise of the corneal cross-linking (CXL) technique in many academic and non-academic clinical settings around the world. Originally developed in Europe, more precisely in Germany and Switzerland, CXL proposed a new therapeutic alternative to patients with progressive keratoconus with the option of stabilizing the disease through a one-time extra-ocular surgical treatment [1, 2]. Easy to perform and efficient after one treatment, this technique became the benchmark for the treatment of progressive keratoconus. With this initial success, the technique was later adapted to treat iatrogenic corneal ectatic disorders [3, 4], bullous keratopathy [5, 6], and melting corneal ulcerations [7]. Initially, only advanced cases of corneal keratitis were treated, but more recently, beginning infections are also responding positively to CXL (Fig. 1, a-d). The number of publications reporting successful treatment of infectious keratitis with CXL is increasing. CXL could become a new alternative to standard treatment of infectious keratitis in the future.

## Review

### CXL for corneal ectasia

CXL was initially developed to treat progressing corneal ectatic disorders such as keratoconus. The idea is to artificially strengthen the cornea biomechanically in order to stop the progression of the disease through the combination of ultraviolet light and a photo-reactant within the corneal stroma. The method was initially developed by Theo Seiler, Eberhard Spoerl, and Gregor Wollensak. Their first report tested this hypothesis ex vivo on porcine corneas and obtained good results, later confirmed in vivo in rabbits and ex vivo on human corneas [2, 8–10]. The first clinical trial was published in 2003 treating 23 eyes with CXL, stabilizing the disease in all patients [1].

CXL was performed using the combination of ultraviolet light type A (UV-A) and vitamin B_2_ (also known as riboflavin). The original Dresden protocol mentions that the epithelium is mechanically removed, followed by application of a 0.1 % riboflavin solution [11]. Once the corneal stroma is soaked with riboflavin, UV-A light (365 nm) is used to irradiate the cornea for 30 min (3 mW/cm^2^ irradiance). Riboflavin is a photo-reacting molecule, which produces free radicals within the corneal stroma when photoactivated. These free radicals interact with the local collagen and proteoglycan molecules to create new covalent bonds and thus strengthen the overall biomechanical resistance of the cornea. Laboratory testing on human corneas reported an increase in corneal rigidity by an average of 300 % after this protocol [2]. Some caution was taken when performing this surgery as only cornea thicker than 400 μm without the epithelium were treated in order to avoid damage to the endothelium [11]. Specific protocols have since been developed to address the issue of thin corneas using a hypo-osmolaric riboflavin solution [12, 13]. This surgery has now been proven to be efficient in the long term with excellent results of disease stabilization over 10 years, and reduces the need of corneal transplant in this population [13–17]. Further, it not only stops the progression of the disease in adults and in children [18–20], but also flattens the keratometry readings in some [1, 16]. Under certain circumstances, however, even CXL fails to stabilize the biomechanically weakened cornea, i.e. in pregnancy [21].

This first accomplishment of halting the progression of keratoconus using CXL led to another application of this technology. CXL was used to treat iatrogenic corneal ectatic disorders developed after laser-assisted in situ keratomileusis (LASIK) and photorefractive keratectomy (PRK) [3, 4]. In clinical trials, CXL stabilized Kmax values, improved corrected distance visual acuity (CDVA), and proved to be an efficient treatment for those rare postoperative complications [3, 4, 22–24].

### A new field to explore

Very early in the history of CXL, another field was explored where the concept of CXL could also be applied. In 2000, Seiler and his team reported the use of CXL for advanced non-infectious corneal melting [25]. This pilot trial treated four patients with corneal melting of various non-infectious origins [25]. The rationale was that an increase in covalent bonds amongst the collagen molecules might help stabilize the disease and avoid any à chaud corneal surgery in these patients. Of four patients, three showed stabilization of the melting process after CXL, whereas the last patient continued to progress despite treatment. From this pilot data, it was concluded that CXL could, in some cases, stabilize the cornea even though a pathological process had already modified its structure.

### PACK-CXL for keratitis

The treatment of advance corneal melting came back into clinical perspective 8 years later with a clinical trial from Iseli and colleagues where therapy-resistant cases of melting corneas were treated with CXL. This time, every case was of infectious origin [7]. In this small cohort study, five patients with advanced corneal melt of infectious origin were selected. Two patients presented a fungal keratitis, whereas the other three were infected with Mycobacterium spp. pathogen. Each patient presented a disease unresponsive to full topical and systemic microbicidal therapy. They received CXL with the standard Dresden protocol technique: 3 mW/cm^2^ CXL for 30 min [2, 7]. After surgery, the melting process was halted in four out of five patients. The last patient showed a persistent corneal melt caused by an immune reaction without any remaining active pathogen. This study not only confirmed the previous results from Seiler and colleagues from the year 2000, but also introduced the concept that CXL might be efficient when treating corneal melts from an infectious origin. Subsequently, further clinical trials on advanced melting corneas, one meta-analysis, and multiple animal experiments confirmed those initial results [20, 26–53].

The next step after treating therapy-resistant cases of advanced infectious keratitis was to treat early cases with CXL. Thus in 2011, Makdoumi and colleagues from Sweden proposed a prospective non-randomized clinical trial to investigate the efficiency of CXL as first line therapy in bacterial keratitis [35]. They recruited 16 patients clinically diagnosed with infectious bacterial keratitis who had not received any previous antibiotic (topical or systemic) treatment. All patients were treated with standard CXL as mentioned in the Dresden protocol (3 mw/cm^2^ for 30 min with 0.1 % riboflavin) [1]. Of 16 patients, 15 showed complete epithelial closure and all showed signs of improvement and reduction of inflammatory response to the bacteria. Nevertheless, two patients needed additional antibiotic therapy to treat their disease [35]. The Swedish team uncovered the microbicidal effect of CXL in early bacterial keratitis as a first-line treatment in this trial.

CXL has been shown to be an efficient treatment stabilizing not only advanced melting corneal ulcers, but also early infectious keratitis of bacterial origin over a 10-year period [26, 37, 41, 44–47, 54]. Photoactivated chromophore for keratitis-corneal collagen cross-linking (PACK-CXL) has a very good healing rate regarding bacteria (average 88 %) as reported in a recent meta-analysis and most of the clinical trials are reporting a successful treatment rate when using PACK-CXL in combination with standard antimicrobial therapy [55]. Regarding fungal infections, healing rates are also good (average 78 %). Some of those infections healed with an additional intervention after PACK-CXL. Healing rate might be different when using PACK-CXL alone and further clinical trials are needed to study this issue. Regarding acanthamoeba keratitis, 10 out of 11 cases healed, five of them with retreatment. Although this meta-analysis reports good healing rates for acanthamoeba keratitis, caution should be taken in monitoring patients as almost half of them needed retreatment [55]. More complicated clinical situations were also addressed in few reports using PACK-CXL to treat combined pathological situations with infectious keratitis such as bullous keratopathy or late-onset infections of a corneal graft [56, 57].

Riboflavin and ultraviolet light have been used for decades to kill pathogens, in combination or separately (surface and water disinfection, blood sterilization prior to transfusion, etc.). Therefore, the microbicidal effect of CXL can be explained through the effect of these two elements. First, ultraviolet (UV) light itself has a strong antimicrobial activity as it directly damages DNA and RNA in microbes such as bacteria, but also viruses, and inhibits microbes from replicating [58–61]. Riboflavin also possesses its own microbicidal effect. When it is photoactivated, it releases reactive oxygen species (ROS) that directly interact with the nucleic acids and cell membranes of the microbe [62, 63]. The combined effect of UV-A light and riboflavin has been shown to be superior than their separate effect, with a 10-fold increased cytotoxicity when compared to UV-A alone [58, 64].

With the increasing number of publications on CXL to treat infectious keratitis, a new terminology regarding this specific use was proposed at the 9th International Cross-Linking Congress in 2013. It aims to distinguish CXL for infections from CXL for corneal ectasia in order to avoid confusion in scientific publications and protocols. The term PACK-CXL: Photo-Activated Chromophore for Keratitis – Corneal Cross-Linking was adopted for CXL when treating infectious keratitis [31].

## Conclusion

CXL was initially developed to stabilize keratoconus. The Dresden protocol was developed after abundant laboratory research including animal trials. This protocol was tailored to only treat cornea with an ectatic disorder and to protect the endothelium, avoiding any other complication. As a safe protocol it was used when applying CXL to other diseases in order to avoid any non-desirable postoperative effect, and most studies continue using it for PACK-CXL as originally established. Our group recently demonstrated that an optimization of the protocol’s parameters allows reducing the treatment time without impairing its killing rate on bacterial strains [43]. Already in the literature, some report successful treatment of accelerated protocols in humans and animals [50, 65]. PACK-CXL follows the Bunsen-Roscoe law of reciprocity and accelerated PACK-CXL could be clinically efficient compared to the standard Dresden protocol with careful modifications of the protocol regarding the pathogen type (bacteria, fungus, amoeba) in order to maintain an appropriate killing rate [50, 65, 66].

PACK-CXL now needs further protocol modifications to tailor the treatment to a specific pathogen or clinical situation. Adjustments of treatment parameters such as irradiation duration, type of chromophore, and fluence used might help increase the microbicidal efficiency of PACK-CXL depending on the type of pathogen. With the possible development of PACK-CXL treating early keratitis and beginning infiltrates, the need for a more cost effective method will be necessary. Cheaper, lighter and smaller devices could support the use of PACK-CXL for early infectious keratitis and help reduce the burden of multi-drug-resistant pathogens and patient’s compliance in those clinical situations.
